# Anal fistula communicating to anterior abdominal wall treated with ksharasutra (medicated seton): a rare case study

**DOI:** 10.1093/jscr/rjaa189

**Published:** 2020-08-15

**Authors:** Ramesh Bhat Punchoor, Deepthirani PN, Sandeep Kiliyankandy, Sathiajith Kizhakkayil

**Affiliations:** 1 Department of Anorectal Diseases, AVP Ayushman Hospital, Kozhikode, Kerala, India; 2 Department of Shalyatantra (Surgery in Ayurveda, Indian Medicine), Sri Sri College of Ayurvedic Science and Research Hospital, Bangalore, India

## Abstract

A case of anal fistula communication to anterior abdominal wall treated using ‘Medicated Seton’ called *‘*Ksharasutra’ is reported here. Sushruta mentioned about Ksharasutra in his text book *Sushruta Samhita* in 500 BC. A 54-year-old male came with recurrent abscess in the lower abdominal wall and underwent incision and drainage three times earlier. Scar on lower abdomen and active sinuses on right and left ischiorectal fossa were noted. Internal opening was felt at anal mucosa posteriorly. Transrectal ultrasonography revealed posterior anal fistula, extending to left gluteum and traced till left ramus. Seton was placed in the tract from 5’o clock position to internal opening at 6’o clock position. Every week Ksharasutra changed for 8 weeks. This healed internal opening, right and left ischiorectal sinuses. Left tract toward groin and lower abdomen wall showed fibrosis. Same was confirmed through review scan. Six years of follow-up has shown no signs of recurrence.

## HISTORY

A 54-year-old male farmer came with complaints of repeated episodes of recurrent abscess in the lower abdominal wall, associated with fever and chills. This required hospitalization, IV antibiotics, IV fluids administration, incision and drainage three times under anesthesia at different hospitals in Kerala, India. Patient is a known diabetic and was under oral hypoglycemic agents. It was diagnosed earlier as diabetic carbuncle.

### Examination

**Figure 1:**
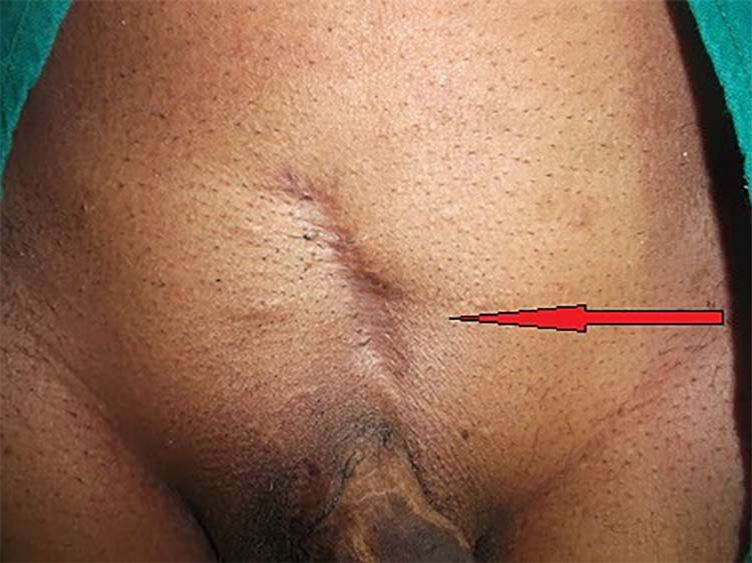
Abdominal scar due to previous surgery in supine position.

There was scar on lower abdomen with mild tenderness with no collection (Fig. 1). There were indurations with an active sinus on right and left ischiorectal fossa. On digital rectal examination, it was found that there was a small induration in lower one-third of anal mucosa at 6’o clock position.

### Investigation

**Figure 2:**
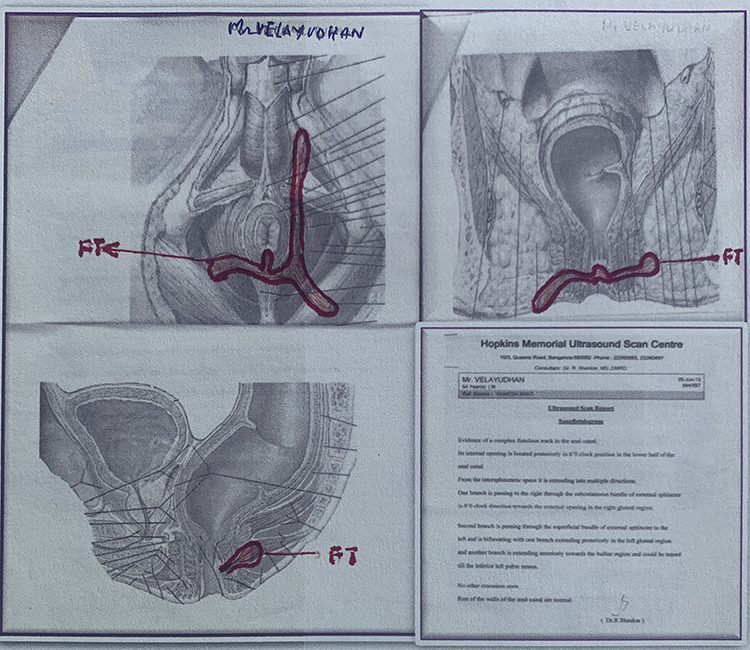
Transrectal scan before Ksharasutra treatment.

**Figure 3:**
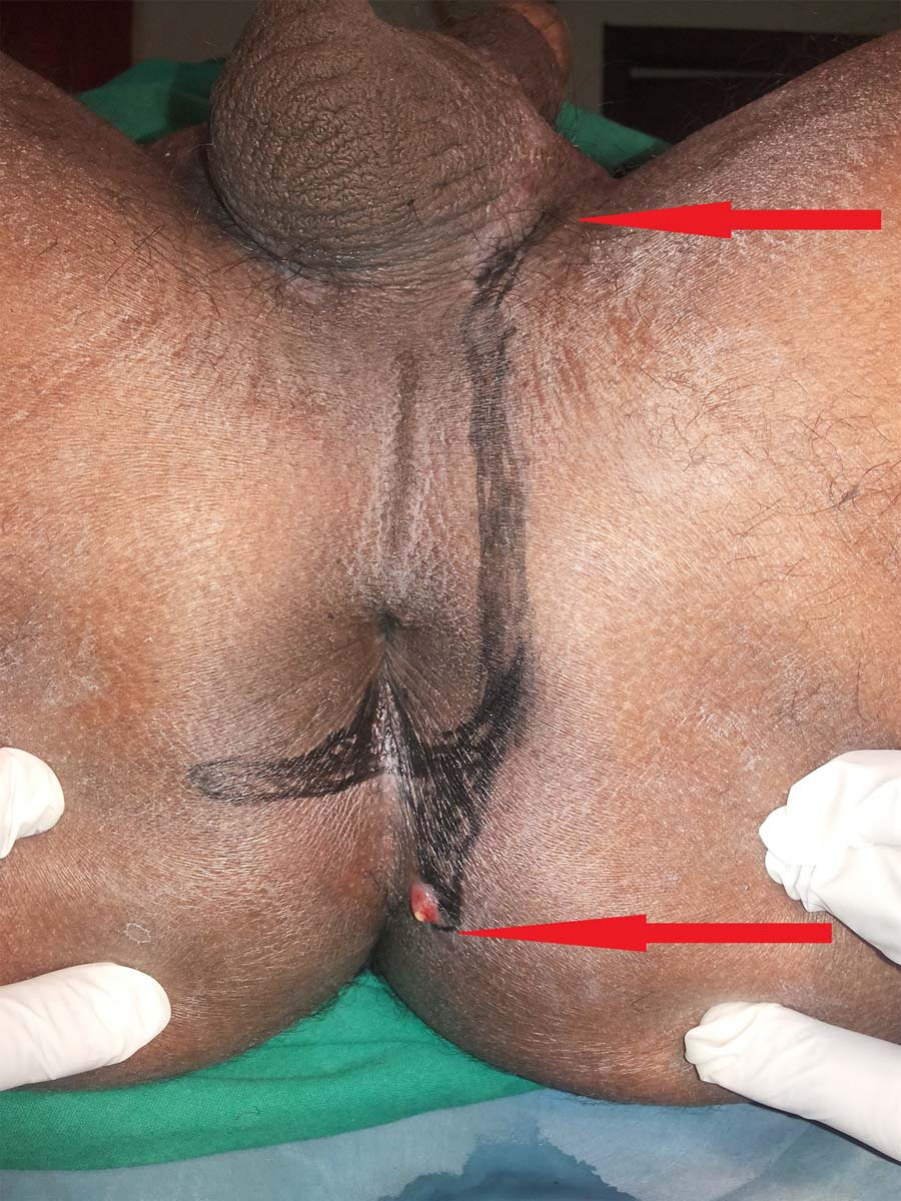
Marking of course of fistula on perianus region of patient in lithotomic position.

Transrectal ultrasonography revealed anal fistula with internal opening at 6’o clock position at lower one-third of anus; from here it coursed downward, one branch toward 8’o clock direction blind with no external opening and another branch toward left gluteum, and superiorly could be traced till left ramus (Figs 2 and 3).

### Procedure/treatment

**Figure 4:**
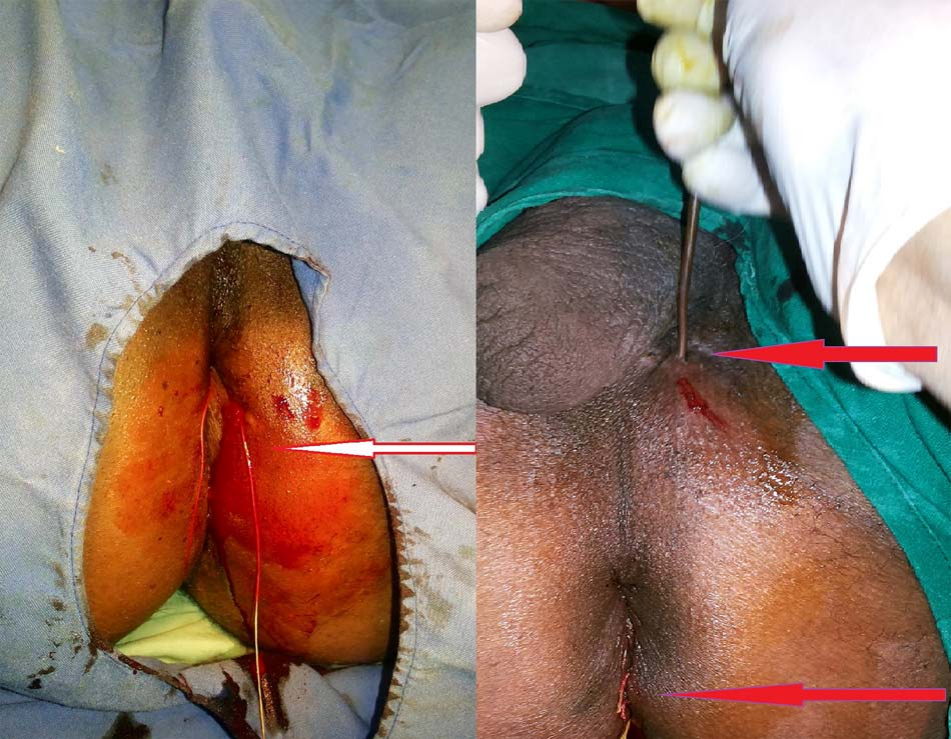
Seton placed to 5' o clock to internal opening and 5' o clock to 11' o clock (base of the scrotum).

Malleable probe passed through external opening at 5’o clock toward lower one-third of anus through internal opening at 6’o clock position. Barbour linen thread No 20 was placed in the tract to sphincter complex (Fig. 4). After 1 week, probe passed through 5’o clock external opening toward left groin till 11’o clock positions near the base of scrotum, and plain Barbour linen thread was placed similarly (Fig. 4). Ksharasutra was changed once every week from 5’o clock to 6’o clock region tract for 8 weeks on outpatient basis.

### Follow-up observation

**Figure 5:**
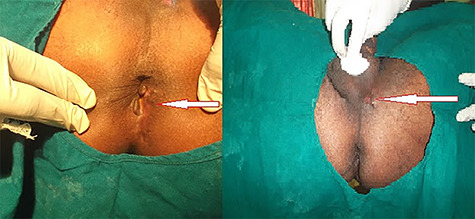
In lithotomic position healed sinus at base of scrotum and ischiorectal fossa.

Tract from 5’o clock to internal opening 6’o clock healed and fibrosed, including internal opening, in 8 weeks of *Ksharasutra*. At sixth week (since its insertion), thread from 5’o clock toward the base of scrotum was also removed with the expectation of healing. At the end of 10th week (since its insertion), the whole tract from 5’o clock toward groin and lower abdomen wall healed showing fibrosis (Fig. 5).

### Review transrectal ultrasonography

The intersphincteric tract and posterior aspect of anus tracts found healed. There was small patent limb of tract from left ischiorectal fossa toward groin found with no signs of fluid in it. Six years of follow-up has shown no recurrence of sinus or abscess in any site along the course of the fistula.

Standard technique of method of preparation of *Ksharasutra*, type of *Ksharasutra*, method of threading and changing of thread was followed as per standard protocol [1] of Banaras Hindu University, Varanasi, India.

## DISCUSSION

Presentation of anal fistula in clinical practice varies and sometimes is difficult to diagnose. Some unusual anal fistulae were reported earlier like fistula communicating to foot [2]. Here, in this case, tract coursing toward anterior abdominal wall was detected. Incision and drainage was done several times previously assuming it to be a diabetic carbuncle. Scar was noted on lower anterior abdominal wall. This may be an extremely rare case ever reported.

*Ksharasutra* treatment for anal fistula has been mentioned in *Sushruta Samhita*, an ancient Indian surgical text book [3, 4]. It is an ideal management for the patients of old age with systemic ailments. No serious side effects are encountered with *Ksharasutra* therapy, although transient infection, local burning sensation, mild pain, itching and slight indurations are observed, which rarely need medication. Postoperative tissue damage and scarring are minimal. The *Ksharasutra* therapy is ambulatory and safe alternative treatment in patients with fistula-in-ano [1].

In *Ksharasutra* therapy, impaired anal continence is minimal [5]. Anal continence is significantly affected after fistula-in-ano surgery, mainly because of sphincteric lesions that affect anal canal pressures and changes in anorectal morphologic and functional parameters after fistula-in-ano surgery [6]. The rate of recurrence [1] of disease in *Ksharasutra* therapy is 3.33%; in conventional surgery it is ~60% [7]

Success rate depends on appropriate and accurate handling of infected anal crypt, rather than fistulectomy or fistulotomy of the entire distal tract or secondary communications. In this case the track extending to right and left tact going toward groin, abdominal wall was neither laid open nor excised. This principle is generally followed in Ksharasutra therapy for fistula with long distal extension [2]. Two centimeters small tract was treated with *Ksharasutra* involving internal opening at 6’o clock position from 5’o clock external opening. *Ksharasutra* was changed without any pain as an outpatient department procedure where patient was doing normal work.

## CONCLUSION

Low-level anal fistula extending to lower abdominal wall was a rare presentation of anal fistula. Therefore, arriving at an accurate diagnosis was difficult. This rare case was managed with *Ksharasutra* therapy, a minimal invasive technique without hospitalization, with the patient doing normal work throughout the treatment. Procedure was done in a minor operation theater setup. There is no incontinence and recurrence when followed up for 6 years.

## CONFLICT OF INTEREST

No conflicts of interest.
